# Long non-coding RNA MALAT1 promotes cardiac remodeling in hypertensive rats by inhibiting the transcription of MyoD

**DOI:** 10.18632/aging.102265

**Published:** 2019-10-15

**Authors:** Dan Li, Chunling Zhang, Jian Li, Jinna Che, Xuecheng Yang, Yuxin Xian, Xueli Li, Caixia Cao

**Affiliations:** 1 Department of Cardiology, The Affiliated Hospital of Qingdao University, Qingdao 266100, P. R. China; 2 Department of Endocrinology, The People’s Hospital of Pingdu, Qingdao 266700, P. R. China; 3 Department of General Medicine, The People's Hospital of Shinan, Qingdao 266002, P. R. China; 4 Department of Endocrinology, The Affiliated Hospital of Qingdao University, Qingdao 266003, P. R. China; 5 Department of Urology Surgery, The Affiliated Hospital of Qingdao University, Qingdao 266003, P. R. China; 6 Department of Geriatrics, The Affiliated Hospital of Qingdao University, Qingdao 266003, P. R. China

**Keywords:** long non-coding RNA MALAT1, MyoD, hypertensive rats, cardiac remodeling

## Abstract

Hypertension is the leading preventable cause of premature deaths worldwide. Although long non-coding RNA (lncRNA) metastasis associated lung adenocarcinoma transcript 1 (MALAT1) has been identified to play important roles in the development of cardiovascular diseases, the regulatory function of lncRNA MALAT1 in hypertension remains poorly understood. This study aimed to explore the role of lncRNA MALAT1 in spontaneously hypertensive rats (SHRs). LncRNA MALAT1 was determined to be elevated and MyoD to be reduced in myocardial tissues and thoracic aortic vascular tissues of SHRs. Over-expression of lncRNA MALAT1 caused severe myocardial fibrosis in SHRs. In addition, lncRNA MALAT1 over-expression *in vitro* enhanced arterial smooth muscle cells (ASMCs) activity and fibrosis of SHRs, which, was rescued by over-expressed MyoD. Furthermore, lncRNA MALAT1 transcripts were found to be highly enriched in the nucleus, and lncRNA MALAT1 suppressed the transactivation of MyoD. Moreover, lncRNA MALAT1 was found to recruit Suv39h1 to MyoD-binding loci, leading to H3K9me3 trimethylation and down-regulation of the target gene. Taken conjointly, this study revealed an important role of lncRNA MALAT1 in promoting cardiac remodeling in hypertensive rats by inhibiting the transcription of MyoD. These results highlight the value of lncRNA MALAT1 as a therapeutic target for the management of hypertension.

## INTRODUCTION

Hypertension is a universal health challenge owing to its high prevalence and resultant cardiovascular disease and chronic kidney disease [1]. Hypertension, as the leading preventable risk factor for disability and premature deaths worldwide, may be attributed to genetic, epigenetic and environmental factors [2, 3]. The most principal mechanism underlying the changes of vascular mechanical property is increased collagen deposition and reduced elastin, which can be also found in hypertension [4]. In addition, the processes of pulmonary vascular remodeling and obliteration of the vessel lumen are implicated in hypertension, which is believed to result in right ventricular failure and premature death [5].

Long non-coding RNAs (lncRNAs) are transcribed genomic regions (longer than 200 nucleotides) without the ability to code for proteins [6]. Metastasis associated lung adenocarcinoma transcript 1 (MALAT1) is a lncRNA that is highly conserved among mammals, and also known as noncoding nuclear-enriched abundant transcript 2 [7]. In human cells, MALAT1 promotes cell proliferation by regulating the transcription and/or post-transcription modification of cell cycle-mediated transcription factors [8]. Similarly, over-expression of MALAT1 promotes cell proliferation and migration in vitro and stimulates tumor growth and metastasis in nude mice with colorectal cancer [9]. Moreover, MALAT1 is highly expressed in human renal cell carcinoma tissues, and MALAT1 silencing resulted in decreased cell proliferation and invasion and increased apoptosis [10]. Microarray-based analysis by Zhang et al*.* revealed that lncRNA MALAT1 was highly expressed in rat models of diabetic cardiomyopathy (DCM), and treatment with silenced lncRNA MALAT1 for 12 weeks caused reduced myocardial apoptosis and improved left ventricle systolic and diastolic functions in DCM [11]. Furthermore, the expression of lncRNA MALAT1 as well as those of inflammatory cytokines including tumor necrosis factor-α, interleukin (IL)-1β, and IL-6 was found to be up-regulated in DCM, and the down-regulation of lncRNA MALAT1 was accompanied by decreased concentration of these cytokines, suggesting that lncRNA MALAT1 is very likely to participate in the inflammatory progression of DCM [12]. In addition, MALAT1 has also been proved to induce pulmonary arterial hypertension susceptibility in Chinese people [13]. More significantly, MALAT1 has been found to be over-expressed in patients with white-coat hypertension [14].

Normally, vascular smooth muscle cells (VSMCs) maintain the vascular contraction function with low activity of proliferation and migration; however, when vascular endothelial injury occurs, VSMCs can potentially switch to a synthetic phenotype with promoted proliferation and migration activity and participate in vascular remodeling and proliferation [15]. The KLF4/MyoD/SRF axis has also been identified as a major molecule axis regulating the phenotypic transition of VSMCs [16]. Recently, it was reported that several microRNAs (miRs) including miR-24, miR-221, and miR-222 could regulate the phenotypic transition of VSMCs and miRs including miR-1, miR-21, miR-143, and miR-145 could modulate the transition of the contractile phenotype by interacting with the KLF4/MyoD/SRF axis [17]. Furthermore, Chen et al. revealed that MALAT1 could inhibit the transactivation of MyoD, which is exclusively expressed in muscle cells and exerts a regulatory role in activating muscle-specific gene expression [18]. MyoD activates the essential early muscle gene Myogenin along with growth arrest and late muscle gene expression [19]. Based on the aforementioned literature, we hypothesized that lncRNA MALAT1 could regulate hypertension by mediating the transcriptional activity of MyoD. In the current study, we aim to demonstrate the important role played by lncRNA MALAT1 on cardiac remodeling in hypertension.

## RESULTS

### LncRNA MALAT1 is highly expressed in myocardial tissues and thoracic aortic vascular tissues of hypertensive rats

**Figure 1:**
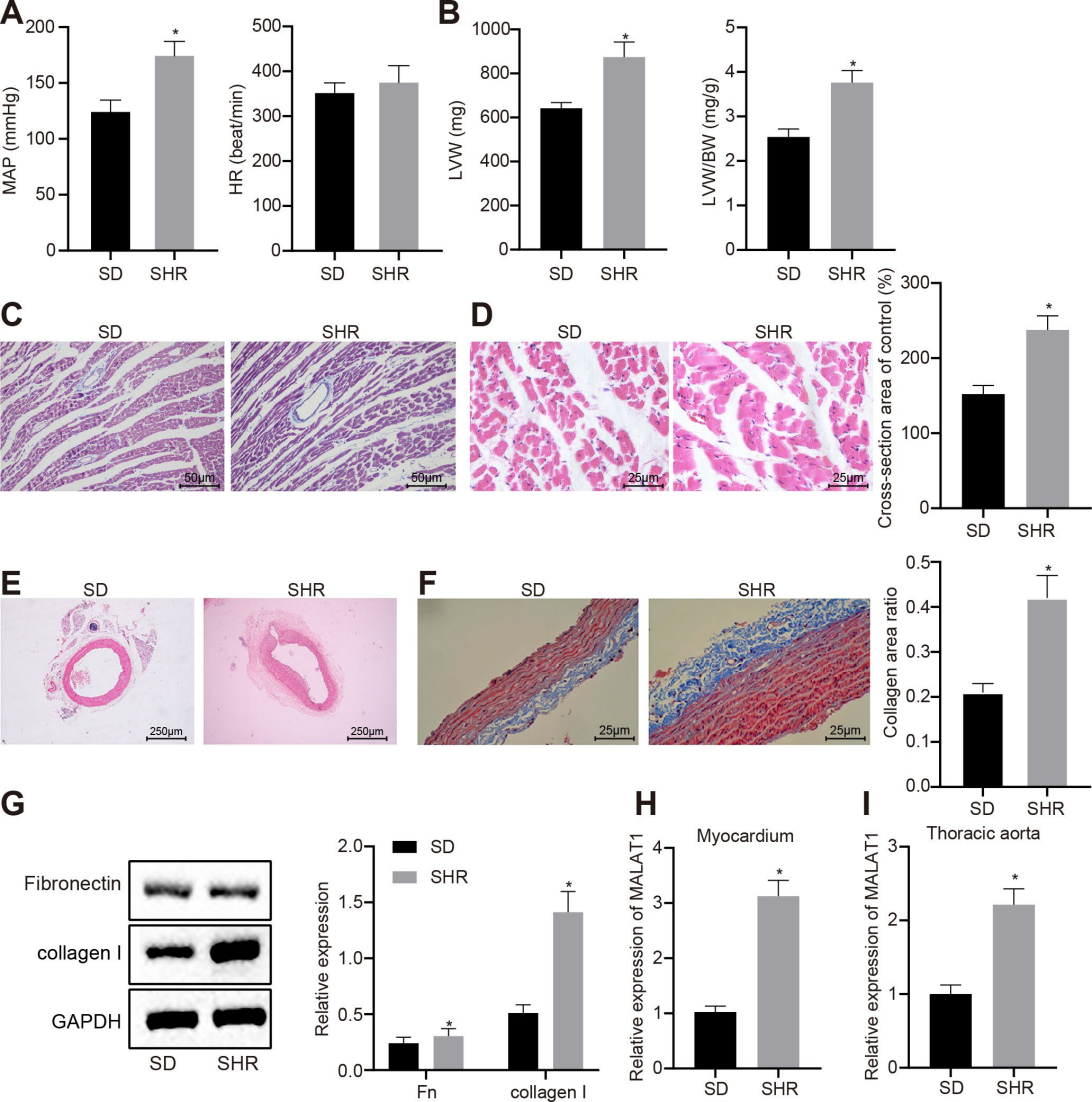
**LncRNA MALAT1 is up-regulated in hypertensive rats.** (**A**) MAP and HR of SD and SHRs; (**B**) LVW and LVW/BW ratio in SD and SHRs; (**C**) images of myocardial tissues observed by Masson staining (scale bar = 50 μm); (**D**) images of myocardial tissues detected by HE staining (scale bar = 25 μm); (**E**) pathological changes of thoracic aortic vascular tissues in SD rats and SHRs observed using HE staining (× 40); (**F**) collagen deposition in thoracic aortic vascular tissues in SD rats and SHRs observed using Masson staining (× 400); (**G**) the expression of fibronectin and collagen I in vascular tissues of SD rats and SHRs, tested by Western blot analysis; (**H**–**I**) the expression of lncRNA MALAT1 in myocardial tissues (**H**) and thoracic aortic vascular tissues (**I**) in SD rats and SHRs, tested by RT-qPCR; *, *p* < 0.05 *vs.* the SD rats; measurement data were expressed by means ± standard deviation and analyzed by unpaired *t-*test; SD rats = 30, SHRs = 10.

We measured the mean arterial pressure (MAP) and heart rate (HR) of Sprague-Dawley (SD) rats and spontaneously hypertensive rats (SHRs) under anesthesia. It was found that the SHRs exhibited higher MAP compared to the SD rats (*p* < 0.05); however, the HR did not differ evidently between the SHRs and SD rats (Figure 1A). Additionally, we evaluated the left ventricle and vascular remodeling in the rats using hematoxylin-eosin (HE) staining and Masson staining, respectively. The results revealed that the left ventricle weight (LVW) and LVW/body weight (BW) in SHRs were significantly higher than those in SD rats and the SHRs presented with mild myocardial fibrosis, severe perivascular fibrosis, hypertrophic myocardial cells, and increased cross-section area of myocardial cells (Figure 1B–1D). Moreover, compared with SD rats, the SHRs had thickened vascular wall of the thoracic aorta and narrowed lumen; obvious collagen (blue) deposition was observed in the thoracic aortic vascular tissues of SHRs (Figure 1E–1F).

Western blot analysis showed that the expression levels of fibronectin and collagen I in vascular tissues of SHRs were significantly elevated in comparison with the SD rats (*p* < 0.05) (Figure 1G), indicating that fibrosis was induced in the vascular tissues of hypertensive rats.

Furthermore, a previous study indicated that lncRNA MALAT1 could induce myocardial fibrosis [20] and pulmonary vascular remodeling of pulmonary artery hypertension [21]; however, the effects of lncRNA MALAT1 on thoracic aorta and myocardial remodeling of hypertensive rats remain to be explored. In order to investigate the role of lncRNA MALAT1 in thoracic aorta and myocardial remodeling of hypertensive rats, we initially detected lncRNA MALAT1 expression using RT-qPCR and the results revealed that lncRNA MALAT1 expression in myocardial tissues and thoracic aorta of SHRs was higher than that in SD rats (Figure 1H–1I). Therefore, we selected SHRs to infect with lentivirus to conduct the subsequent experiments.

### Over-expression of lncRNA MALAT1 promotes cardiac remodeling in hypertensive rats

**Figure 2:**
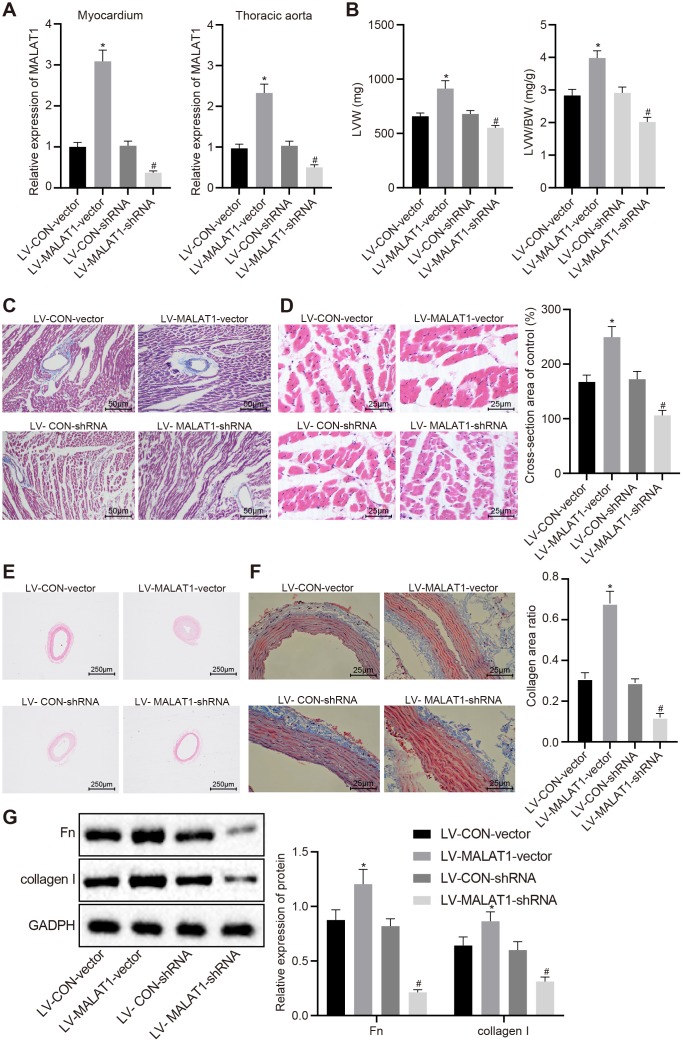
**LncRNA MALAT1 over-expression promotes cardiac remodeling in hypertensive rats.** (**A**) the expression of lncRNA MALAT1 in myocardial tissues and thoracic aortic vascular tissues of SHRs after over-expression or silencing of lncRNA MALAT1 detected by RT-qPCR; B, LVW and LVW/BW ratio in SHRs after over-expression or silencing of lncRNA MALAT1; (**C**) Masson staining images of myocardial tissues after over-expression or silencing of lncRNA MALAT1 (scale bar = 50 μm); (**D**) HE staining images of myocardial tissues after over-expression or silencing of lncRNA MALAT1 (scale bar = 25 μm); (**E**) pathological changes of thoracic aortic vascular tissues of SHRs observed using HE staining after over-expression or silencing of lncRNA MALAT1 (× 40); (**F**) collagen deposition in thoracic aortic vascular tissues of SHRs after over-expression or silencing of lncRNA MALAT1, observed by Masson staining (× 400); (**G**) the expression of fibronectin and collagen protein in thoracic aortic vascular tissues of SHRs after over-expression or silencing of lncRNA MALAT1, determined by Western blot analysis; *, *p* < 0.05 *vs.* the LV-CON-vector group; #, *p* < 0.05 *vs.* the LV-CON-shRNA group; measurement data were expressed by means ± standard deviation and analyzed by one-way analysis of variance; n = 6.

In order to explore the effect of lncRNA MALAT1 on cardiac remodeling in hypertensive rats, we injected lentivirus over-expressing or silencing lncRNA MALAT1 into the SHRs and subsequently detected the expression of lncRNA MALAT1 in myocardial tissues and thoracic aortic vascular tissues. The results revealed showed that compared with the SHRs in the lentiviral vector (LV)-CON-vector group, the expression of lncRNA MALAT1 was upregulated in myocardial tissues and thoracic aortic vascular tissues of SHRs in the LV-MALAT1-vector group. When compared to the SHRs in the LV-CON-shRNA group, the expression of lncRNA MALAT1 was down-regulated in myocardial tissues and thoracic aortic vascular tissues of SHRs in the LV-MALAT1-shRNA group (Figure 2A). In addition, we measured the LVW and LVW/BW ratio of SHRs and found that over-expressing lncRNA MALAT1 resulted in increased LVW and LVW/BW ratio, while silencing lncRNA MALAT1 caused the opposite results (Figure 2B). Moreover, HE staining and Masson staining of myocardial tissues demonstrated that lncRNA MALAT1 over-expression led to more severe myocardial fibrosis and more hypertrophic myocardial cells, which were opposite to the trend caused by lncRNA MALAT1 silencing (Figure 2C–2D). Furthermore, HE staining and Masson staining of the thoracic aortic vascular tissues of SHRs revealed that the vascular walls of thoracic aorta in rats were thickened, the lumen of blood vessel was narrowed, collagen (blue) deposition was elevated in response to lncRNA MALAT1 over-expression, whereas aortic remodeling was attenuated following lncRNA MALAT1 silencing (Figure 2E–2F). Western blot analysis results (Figure 2G) revealed that the expression of fibronectin and collagen I in vascular tissues of rats in the LV-MALAT1-vector group was significantly elevated compared to the LV-CON-vector group (*p* < 0.05). Whereas compared with the LV-CON-shRNA group, the levels of fibronectin and collagen I in vascular tissues of the LV-MALAT1-shRNA group were significantly decreased (*p* < 0.05). These findings evidenced that cardiac remodeling in hypertensive rats is promoted by over-expression of lncRNA MALAT1.

### Over-expression of lncRNA MALAT1 enhances cell proliferation and fibrosis of arterial smooth muscle cells (ASMCs)

**Figure 3:**
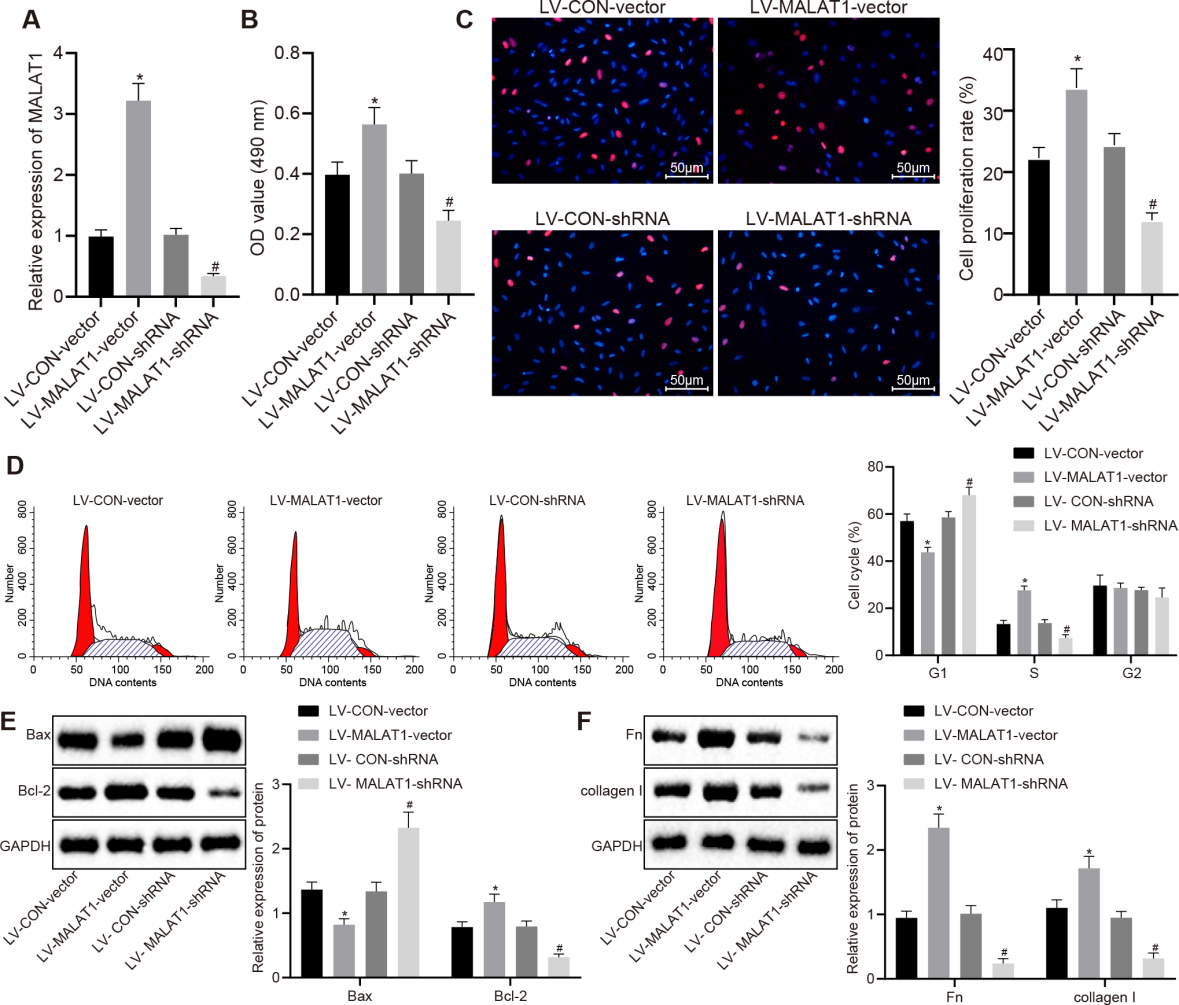
**Over-expression of lncRNA MALAT1 enhances ASMC proliferation and fibrosis.** (**A**) expression of lncRNA MALAT1 in ASMCs of SHRs after over-expression or silencing of lncRNA MALAT1 detected using RT-qPCR; (**B**) cell activity after over-expression or silencing of lncRNA MALAT1, determined by MTT assay; (**C**) proliferation of ASMCs after over-expression or silencing of lncRNA MALAT1, measured by BrdU assay (× 200); (**D**) cell cycle analysis after over-expression or silencing of lncRNA MALAT1, determined by flow cytometry; (**E**) the protein expression of Bcl-2 and Bax in ASMCs after over-expression or silencing of lncRNA MALAT1 as determined by Western blot analysis; (**F**) the expression of fibronectin and collagen I in ASMCs after over-expression or silencing of lncRNA MALAT1, detected by Western blot analysis; *, *p* < 0.05 *vs.* the LV-CON-vector group; #, *p* < 0.05 *vs.* the LV-MALAT1-vector group; measurement data were expressed by means ± standard deviation; data in panel (**A**–**C**, **E** and **F**) were analyzed by one-way analysis of variance; data in panel (**D**) were analyzed by repeated-measures analysis of variance; n = 3.

ASMCs were isolated from SHRs and infected with lentivirus over-expressing or silencing lncRNA MALAT1 and the expression of lncRNA MALAT1 after lentivirus infection was detected using reverse transcription quantitative polymerase chain reaction (RT-qPCR). The results showed that the lncRNA MALAT1 expression was elevated in ASMCs in the LV-MALAT1-vector group compared to the LV-CON-vector group; when relative to the LV-CON-shRNA group, the LV-MALAT1-shRNA group exhibited reduced expression of lncRNA MALAT1 (Figure 3A). Subsequently, cell activities and proliferation of ASMCs in SHRs after lentivirus infection were determined by 3-(4, 5-Dimethylthiazol-2-yl)-2, 5-diphenyltetrazolium bromide (MTT) assay and Bromodeoxyuridine (BrdU) assay, respectively. As shown in Figure 3B and 3C, the cell activity and proliferation rate in the LV-MALAT1-vector group were significantly increased compared to the LV-CON-vector group (*p* < 0.05). Whereas, compared with the LV-CON-shRNA group, the cell activity and proliferation rate in the LV-MALAT1-shRNA group rats were significantly decreased (*p* < 0.05). The results of cell cycle detected using flow cytometry displayed in Figure 3D further verified the abovementioned results. Compared with the LV-CON-vector group, cells at the S phase were significantly increased in the LV-MALAT1-vector group (*p* < 0.05). Compared with the LV-CON-shRNA group, cells at the S phase were significantly decreased in the LV-MALAT1-shRNA group (*p* < 0.05). Moreover, Western blot analysis results also revealed that the protein expression of Bcl-2 was up-regulated, while that of Bax was down-regulated in the LV-MALAT1-vector group versus the LV-CON-vector group, which was opposite to the trends observed in the LV-MALAT1-shRNA group relative to the LV-CON-shRNA group (all *p* < 0.05) (Figure 3E).

Additionally, the expression of fibronectin and collagen I in ASMCs of SHRs was analyzed by Western blot analysis. As shown in Figure 3F, the expression of fibronectin and collagen I in LV-MALAT1-vector group was significantly increased compared to the LV-CON-vector group. Whereas, compared with the LV-CON-shRNA group, the expression of fibronectin and collagen in the LV-MALAT1-shRNA group was significantly decreased (*p* < 0.05). The abovementioned results and findings indicated that over-expression of lncRNA MALAT1 promotes cell proliferation and fibrosis of ASMCs.

### LncRNA MALAT1 inhibits the transcription of MyoD

**Figure 4:**
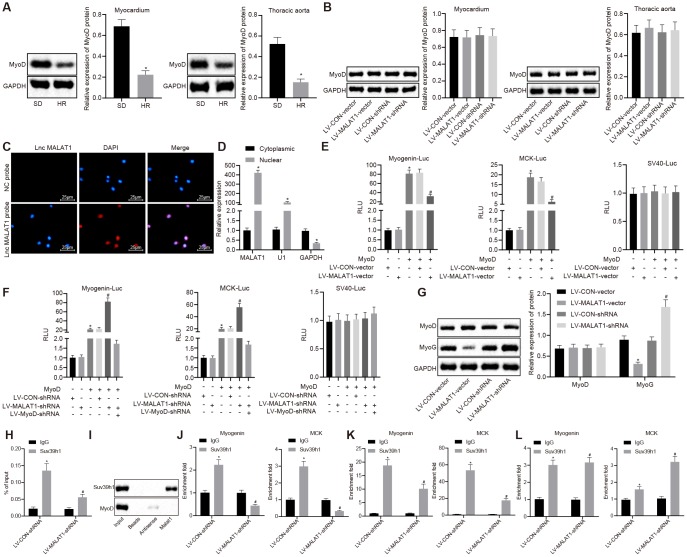
**LncRNA MALAT1 over-expression suppresses the transcription of MyoD.** (**A**) the expression of MyoD in myocardial tissues and thoracic aortic vascular tissues of SD rats and SHRs detected by Western blot analysis; (**B**) the expression of MyoD in myocardial tissues and thoracic aortic vascular tissues of SHRs after over-expression or silencing of lncRNA MALAT1; (**C**) cellular location of lncRNA MALAT1 determined using FISH (× 400); (**D**) enrichment of lncRNA MALAT1, U1, and GAPDH in nuclear or cytoplasmic fractions of ASMCs; (**A**) luciferase activity of Myogenin-Luc and MCK-Luc in VMSCs in response to over-expression or silencing of lncRNA MALAT1; (**G**) the expression of MyoD and MyoG in VSMCs of SHRs after over-expression or silencing of lncRNA MALAT1, as measured by Western blot analysis; (**H**) RIP assay of nuclear extracts from the VSMCs immunoprecipitated by IgG or an antibody against Suv39h1 and RT-qPCR measurement of the retrieved RNAs; (**I**) RNA pull-down assay of in vitro-transcribed biotinylated full-length lncRNA MALAT1 transcripts and the binding proteins as well as Western blot analysis of the indicated proteins; (**J**–**L**) ChIP-PCR analysis of Suv39h1, H3K9me3 and MyoD enrichment on the promoter or enhancer of Myogenin, MCK loci in VSMCs. The enrichment fold was calculated as a fraction of DNA present in the input samples. *, *p* < 0.05 *vs.* the SD rats or cytoplasmic RNA or LV-CON-vector group or LV-CON-shRNA group or IgG group; #, *p* < 0.05 *vs.* the MyoD or Suv39h1 or H3K9me3; measurement data were expressed by means ± standard deviation and analyzed by unpaired *t-*test; n = 3.

LncRNA MALAT1 possesses the ability to suppress the transcriptional activity of MyoD [22], a process which has been previously associated with vascular remodeling [23]. Therefore, in order to explore whether lncRNA MALAT1 in myocardial tissues and thoracic aortic vascular tissues regulates the transcription of MyoD to affect cardiac remodeling in hypertensive rats, we analyzed the expression of MyoD in myocardial tissues and thoracic aortic vascular tissues of SD rats and SHRs. It was determined that SHRs presented with lower expression of MyoD in myocardial tissues and thoracic aortic vascular tissues compared to SD rats (Figure 4A), which was reverse to lncRNA MALAT1 expression. In addition, the expression of MyoD in myocardial tissues and thoracic aortic vascular tissues of SHRs after over-expressing or silencing lncRNA MALAT1 was also detected. The results revealed that there was no obvious difference concerning the MyoD expression in myocardial tissues and thoracic aortic vascular tissues of SHRs after over-expressing or silencing lncRNA MALAT1 (Figure 4B). Hence, we assumed that lncRNA MALAT1 might regulate the expression of target gene by influencing the transcriptional activity of MyoD, thus promoting cardiac remodeling.

Subsequently, we performed several assays in order to investigate whether lncRNA MALAT1 could repress the transcriptional activity of MyoD. First, subcellular localization of lncRNA MALAT1 in ASMCs of SHRs was determined using fluorescence in situ hybridization (FISH), which revealed that lncRNA MALAT1 was solely located in the nucleus (Figure 4C). Second, results of fractionation of nuclear/cytoplasmic RNA showed that lncRNA MALAT1 transcript was highly enriched in the nucleus and the existence of U1 was detected; also, it was found that glyceraldehyde phosphate dehydrogenase (GAPDH) transcript primarily existed in the cytoplasmic RNA (Figure 4D), suggesting that lncRNA MALAT1 was mainly expressed in the nucleus. Third, with the expression of lncRNA MALAT1 over-expressed or depleted, the VSMCs were co-transfected with MyoD expression plasmid and MyoD luciferase reporter (Myogenin-Luc or MCK-Luc). As revealed by dual-luciferase reporter assay, lncRNA MALAT1 over-expression inhibited the MyoD activation of these reporters (Figure 4E), while lncRNA MALAT1 silencing induced the MyoD activation of these reporters; however, after the expression of MyoD was knocked down, lncRNA MALAT1 silencing failed to result in MyoD activation of these reporters (Figure 4F). At last, we measured the expression of MyoD in VSMCs and the results indicated that the expression of MyoD did not differ in response to over-expression or silencing of lncRNA MALAT1 (Figure 4G). These results suggested that lncRNA MALAT1 inhibited the transactivation of MyoD but did not affect the expression of MyoD.

Additionally, we explored how lncRNA MALAT1 represses the transactivation ability of MyoD. We initially speculated that lncRNA MALAT1 might recruit an inhibitory cofactor to the MyoD binding site. Since lncRNA MALAT1 could interact with Set domain-containing proteins [24], we speculated that lncRNA MALAT1 could interact with Suv39h1 and recruit Suv39h1 to MyoD. Furthermore, it has been reported that histone methyltransferase Suv39h1 inhibits the transcriptional activity of MyoD [25]. To verify our speculation, we performed RNA binding protein immunoprecipitation (RIP) assay and found that the anti-Suv39h1 protein antibody captured a large number of endogenous lncRNA MALAT1 transcripts from VSMCs (Figure 4H), which indicated the existence of a presumptive interaction between Suv39h1 protein and lncRNA MALAT1 transcripts. To better demonstrate this finding, we conducted RNA pull-down using the biotinylated full-length lncRNA MALAT1 transcript in natural uncross-linked cell lysates. Consistent with the results of RIP assay, the results of RNA pull-down demonstrated that lncRNA MALAT1 transcripts pulled down a large number of endogenous Suv39h1 proteins, while not pulling down the MyoD protein (Figure 4I), suggesting that the physical interaction existed between Suv39h1 and lncRNA MALAT1, but did not exist between MyoD and lncRNA MALAT1. Moreover, the Suv39h1 protein was not retrieved in negative control (NC) bead and the antisense MALAT1 transcript (Figure 4I).

Subsequently, in order to elucidate whether lncRNA MALAT1 could tether to the MyoD-binding DNA loci, we applied chromatin immunoprecipitation (ChIP) assay, whose results proved that silencing of lncRNA MALAT1 repressed the binding of endogenous Suv39h1 protein and MyoD loci on the Myogenin and MCK promoter or enhancer (Figure 4J), which confirmed that lncRNA MALAT1 played a significant role in the recruitment of Suv39h1 to MyoD loci. Unexpectedly, lncRNA MALAT1 silencing resulted in reduced expression of H3K9me3 on the above loci (Figure 4K). Interestingly, it was also found that silencing of lncRNA MALAT1 promoted the enrichment of MyoD on Myogenin and MCK (Figure 4L).

Collectively, the abovementioned findings provided evidence demonstrating that lncRNA MALAT1 recruits Suv39h1 to MyoD-binding loci, thus inducing trimethylation of H3K9me3 and inhibiting the expression of the target gene.

### MyoD over-expression reverses the promoting effects of lncRNA MALAT1 and attenuates cardiac remodeling in hypertensive rats

**Figure 5:**
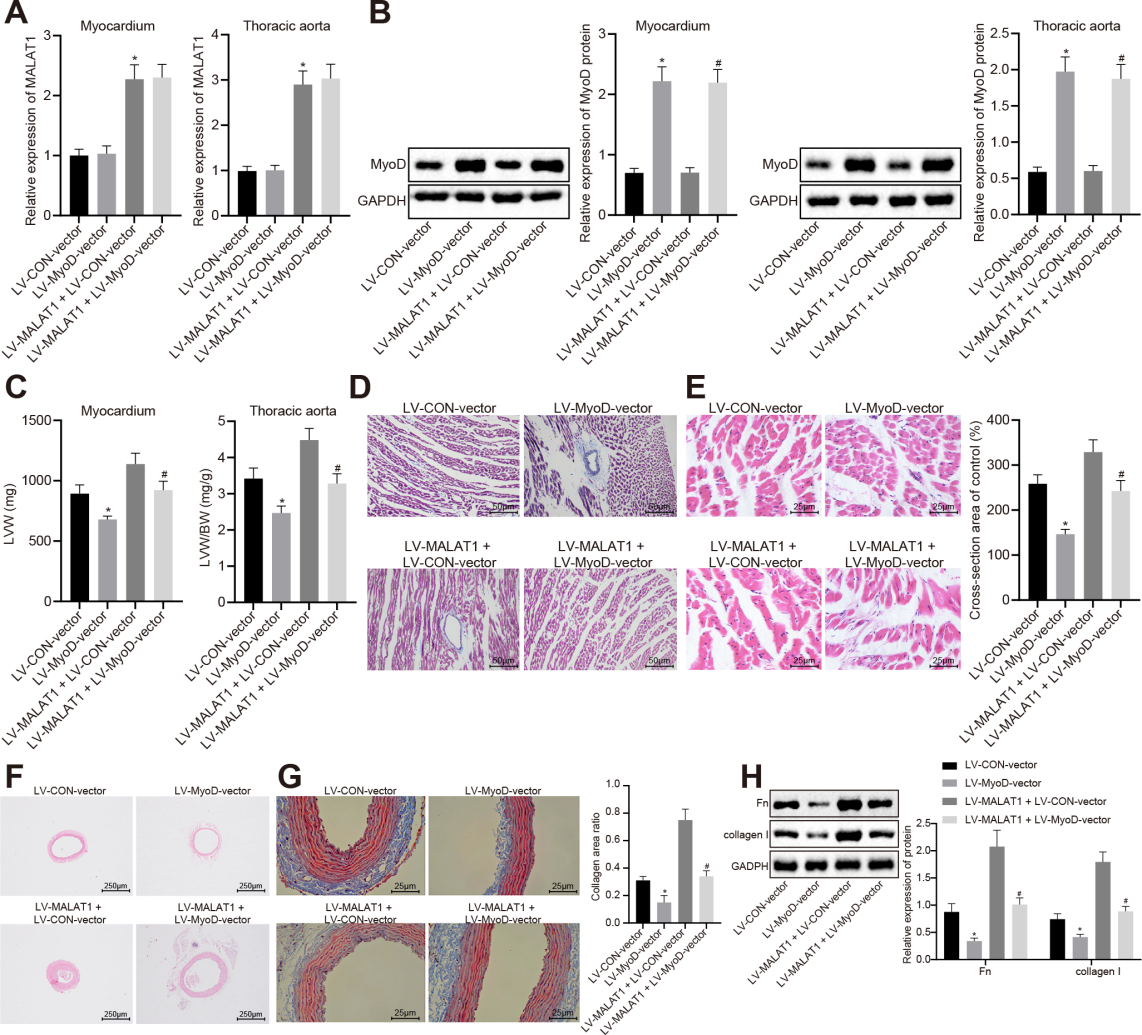
**The promoting effects of over-expressed lncRNA MALAT1 on cardiac remodeling in hypertensive rats is reversed by over-expression of MyoD.** (**A**) the expression of lncRNA MALAT1 in myocardial tissues and thoracic aortic vascular tissues of SHRs, as measured by RT-qPCR; (**B**) the expression of MyoD in myocardial tissues and thoracic aortic vascular tissues of SHRs measured using Western blot analysis; (**C**) LVW and LVW/BW ratio of SHRs; (**D**) Masson staining images of myocardial tissues (scale bar = 50 μm); (**E**) HE staining images of myocardial tissues (scale bar = 25 μm); (**F**) pathological changes of thoracic aortic vascular tissues of SHRs, observed by HE staining (× 40); (**F**) collagen deposition in thoracic aortic vascular tissues of SHRs, observed by Masson staining (× 400); (**H**) the expression of fibronectin and collagen I in thoracic aortic vascular tissues of SHRs, measured by Western blot analysis; *, *p* < 0.05 *vs.* the LV-CON-vector group; #, *p* < 0.05 *vs.* the LV-MALAT1 + LV-CON-vector group; measurement data were expressed by means ± standard deviation and analyzed by one-way analysis of variance; n = 6.

In order to explore the involvement of MyoD in vivo, we initially carried our RT-qPCR and Western blot analysis to measure the expression of lncRNA MALAT1 and MyoD in myocardial tissues and thoracic aortic vascular tissues of SHRs. The results showed that compared with the LV-CON-vector group, the expression of lncRNA MALAT1 in myocardial tissues and thoracic aortic vascular tissues of SHRs did not differ significantly, while the expression of MyoD was elevated in the LV-MyoD-vector group, which was consistent with the trends observed in the LV-MALAT1 + LV-MyoD-vector group when in comparison with the LV-MALAT1 + LV-CON-vector group (Figure 5A–5B). In addition, we measured the LVW and LVW/BW ratio of the SHRs and found that over-expressed MyoD led to reduced LVW and LVW/BW and could reverse the promoted effects of LV-MALAT1 on LVW and LVW/BW (Figure 5C). Moreover, the myocardial tissues of rats were analyzed using HE staining and Masson staining and the results showed that myocardial fibrosis and myocardial cell hypertrophy were both attenuated in response to over-expression of MyoD, which could reverse the enhancing role of lncRNA MALAT1 (Figure 5D–5E). Furthermore, cardiac remodeling was observed by HE and Masson staining, and the expression of fibronectin and collagen I in vascular tissues of rats was detected using Western blot analysis. It was observed that the vascular wall of thoracic aorta was thinned, the lumen was widened, the deposition of collagen (stained with blue) was significantly decreased, and the expression of fibronectin and collagen I was significantly decreased following over-expression of MyoD and the effects of lncRNA MALAT1 could be reversed by over-expressed MyoD (Figure 5F–5H). Overall, these findings demonstrated that MyoD could block the effects of lncRNA MALAT1 to inhibit cardiac remodeling in hypertensive rats.

### MyoD over-expression reverses the effects of lncRNA MALAT1 and represses cell proliferation and fibrosis in ASMCs of hypertensive rats

**Figure 6:**
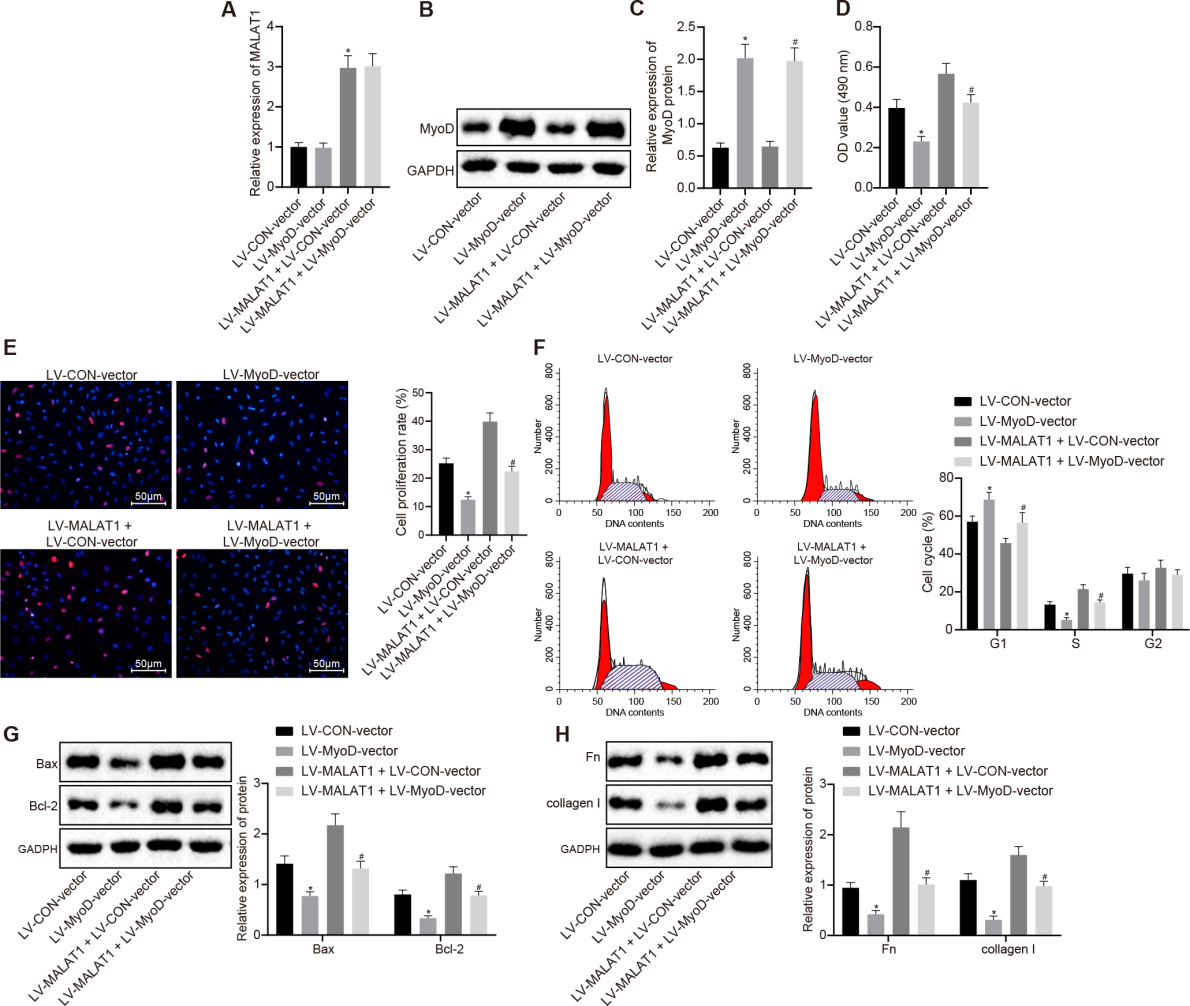
**Over-expression of lncRNA MALAT1 promotes cell proliferation and fibrosis, which, whereas, is reversed by up-regulating MyoD.** (**A**) the expression of lncRNA MALAT1 in ASMCs of SHRs, detected by RT-qPCR; (**B**–**C**) the expression of MyoD in ASMCs of SHRs, detected by Western blot analysis; (**D**) ASMC activity in SHRs, evaluated using MTT assay; (**E**) proliferation of ASMCs in SHRs, determined by BrdU assay (× 200); (**F**) cell cycle analysis in SHRs, determined by flow cytometry; (**G**) the expression of Bax and Bcl-2 in ASMCs of SHRs detected using Western blot analysis; (**H**) the expression of fibronectin and collagen I in ASMCs, tested by Western blot analysis; *, *p* < 0.05 *vs.* the LV-CON-vector group; #, *p* < 0.05 *vs.* the LV-MALAT1 + LV-CON-vector group; measurement data were expressed by means ± standard deviation; data in panel (**A**–**E**, **G** and **H**) were analyzed by one-way analysis of variance; data in panel (**F**) were analyzed by repeated-measures analysis of variance; n = 3.

RT-qPCR and Western blot analysis were conducted to determine the expression of lncRNA MALAT1 and MyoD in ASMCs of SHRs and the results showed that compared with the LV-CON-vector group, the expression of lncRNA MALAT1 did not differ evidently, while that of MyoD was increased in ASMCs in the LV-MyoD-vector group, which was in line with the trend observed in the LV-MALAT1 + LV-MyoD-vector group when compared to the LV-MALAT1 + LV-CON-vector group (Figure 6A–6C). Additionally, cell activity was detected using MTT assay and it was found that the cell activity in the LV-MyoD-vector group was significantly decreased compared to LV-CON-vector group (*p* < 0.05). Compared with LV-MALAT1 + LV-CON-vector group, the cell activity in the LV-MALAT1 + LV-MyoD-vector group was significantly decreased (*p* < 0.05) (Figure 6D). In addition, BrdU assay was employed to measure the cell proliferation of ASMCs of SHRs, and the results indicated that the proliferation rate was attenuated in the LV-MyoD-vector group versus the LV-CON-vector group; in contrast to the LV-MALAT1 + LV-CON-vector group, the proliferation rate was decreased in the LV-MALAT1 + LV-MyoD-vector group (*p* < 0.05) (Figure 6E). Furthermore, flow cytometry results for cell cycle detection further verified the above results (Figure 6F). Moreover, Western blot analysis results revealed that relative to the LV-CON-vector group, the LV-MyoD-vector group presented with reduced protein expression of Bcl-2 and elevated protein expression of Bax, which was concurred with the results found in the LV-MALAT1 + LV-MyoD-vector group in comparison with the LV-MALAT1 + LV-CON-vector group (*p* < 0.05) (Figure 6G). Further, the results of Western blot analysis suggested that the expression of fibronectin and collagen I in the LV-MyoD-vector group was significantly decreased compared to the LV-CON-vector group (*p* < 0.05). Compared with the LV-MALAT1 + LV-CON-vector group, the expression of fibronectin and collagen I in the LV-MALAT1 + LV-MyoD-vector group was significantly decreased (*p* < 0.05) (Figure 6H). The aforementioned results indicated that promoting effects of over-expressed lncRNA MALAT1 on cell proliferation and fibrosis of hypertensive rats could be reversed by MyoD over-expression.

## DISCUSSION

Even though the signaling pathways and the underlying mechanisms that mediate VSMCs phenotype in hypertension have been principally clarified, the controlling function of lncRNAs in hypertension still remains to be elucidated [26]. The current study aimed to explore the role of lncRNA MALAT1 in cardiac remodeling in hypertensive rats. Our findings highlighted the important role of lncRNA MALAT1 in promoting cardiac remodeling in hypertensive rats by inhibiting the transcription of MyoD.

Initially, we found that in hypertensive rats, lncRNA MALAT1 was highly expressed in myocardial tissues and thoracic aortic vascular tissues of hypertensive rats. LncRNAs possess the ability to regulate gene expression through multiple approaches, such as miR sponging, X chromosome inactivation, and chromatin reprogramming [27]. In recent years, lncRNA MALAT1 has been identified to be associated with tumorigenic conditions, including lung cancer, pancreatic cancer, and cervical cancer [28]. Consistent with our study, a previous study identified significantly increased levels of lncRNA MALAT1 in the serum of coronary atherosclerotic heart disease patients [29]. In addition, another study revealed lncRNA MALAT1 was over-expressed in patients with white-coat hypertension [14]. Han et al. demonstrated that the macrophages in diabetic atherosclerosis rats exhibited highly expressed expression of MALAT1, along with elevated systemic inflammatory cytokine production, implying that lncRNA MALAT1 was involved with the biological processes of diabetic atherosclerosis [30]. Furthermore, lncRNA MALAT1 depletion is known to promote VSMC proliferation and left ventricle function, as well as attenuate cardiomyocyte apoptosis, all of which are linked to the development of atherosclerotic heart disease, the induction of which was found to be hindered by the rs19586AG/GG genotype of MALAT1 [31]. Importantly, our study further demonstrated that lncRNA MALAT1 inhibited transcription of MyoD. Similar to our findings, lncRNA MALAT1 has been demonstrated to exert inhibitory effects on the transcription of MyoD in myogenic differentiation [22]. MyoD belongs to the MyoD gene class which is well-known to play critical roles in muscle development and growth [32, 33]. MyoD was found to be decreased in soleus and extensor digitorumlongus muscles in rats suffering from heart failure, which was reported to be correlated with diaphragm myopathy [34, 35].

Additionally, our findings revealed that over-expression of lncRNA MALAT1 promoted the expression of fibronectin and collagen I in vascular tissues of SHRs. Previous studies have highlighted that fibronectin produced from bronchial smooth muscle cells, is implicated in the process of airway remodeling [36]. In addition, lncRNA MALAT1 inhibition has been associated with down-regulation of fibronectin in bladder cancer cells [37]. Collagen, found in arterial walls, is secreted by fibroblasts and smooth muscle cells, and variations in the amount of collagen can lead to the pathogenesis of vascular disease [38]. Furthermore, Castoldi et al. demonstrated that collagen 1a1, whose expression was elevated in response to myocardial fibrosis, could be targeted by miR-133a in myocardial remodeling [39]. Interestingly, lncRNA MALAT1 down-regulation has also been previously demonstrated to exert inhibitory effects on both hepatic stellate cells activation in vitro and collagen deposition in vivo [40].

Moreover, we also found that over-expression of lncRNA MALAT1 promoted cell proliferation, myocardial fibrosis, and cell cycle entry by inhibiting the transcription of MyoD. Consistent with our results, a previous study revealed that lncRNA MALAT1 plays a significant role during myoblast differentiation, wherein knockdown of lncRNA MALAT1 accelerates the myogenic process [22]. Besides, a study exploring the role of lncRNA MALAT1 in the phenotype switching of VSMCs reported that lncRNA MALAT1 silencing suppresses cellular proliferation and migration, resulting in significant cell cycle arrest at the G2 phase [41]. Furthermore, Huang et al. recently demonstrated that lncRNA MALAT1 negatively regulates miR-145 to increase TGF-β1 activity, thus enhancing cardiac fibrosis and deteriorating myocardial infarction-induced cardiac function in mice [20].

Overall, the current study evidenced that lncRNA MALAT1 promotes cardiac remodeling in hypertensive rats by inhibiting the transcription of MyoD. Our findings highlight the potential avenues that lncRNA MALAT1 holds for future development of therapeutic strategies for hypertension. However, additional efforts are necessary to investigate whether lncRNA MALAT1 regulates MyoD activity during hypertension via modulation of TGF-β1 activity or other molecular mechanisms to successfully elucidate the therapeutic use of lncRNA MALAT1.

## MATERIALS AND METHODS

### Ethical statement

The current study was carried out in strict accordance with the recommendations in the Guide for the Care and Use of Laboratory Animals of the National Institutes of Health. All study protocols were approved by the Institutional Animal Care and Use Committee of the Affiliated Hospital of Qingdao University.

### Experimental animals

Fifty-five male SHRs and thirty male SD healthy rats (aged 10 weeks; weighing 250 - 300 g) were purchased from Weitonglihua experimental animal technology Co., Ltd., (Beijing, China) and Qingdao Laboratory Animal Center of the Institute for Drug Control (Qingdao, Shandong, China), respectively. The mean calculated body weight of SHRs was (278 ± 11) g, and that of SD rats was (279 ± 13) g. Ten SHRs and thirty SD rats were selected for blood pressure determination, HE staining and Masson staining.

### Separation and culture of ASMCs

Firstly, 3 SHRs were selected and euthanized by intraperitoneal injection with pentobarbital sodium (150 mg/kg) on an aseptic operating table [42]. Then, the cardiopulmonary tissues were quickly obtained and soaked in aseptic Hanks solution containing penicillin-streptomycin (15140-122, Gibco, Carlsbad, CA, USA). Under the operating microscope, the thoracic artery was swiftly isolated, and the fibrous layer and adventitia were divested. After the endothelial cells were removed using ophthalmic scissors, ASMCs were cultured in a flip culture bottle. After being cultured and purified to the 4^th^ generation, the cells were confirmed by immunohistochemistry and used for the subsequent experiments.

### Grouping and transfection

Lentivirus was prepared and packed by the Shanghai Genechem Co., Ltd. (Shanghai, China). The remaining 42 SHRs and AMSCs were respectively assigned into the following 7 groups (6 rats in each group): the LV-CON-vector group (infected with lentivirus over-expressing empty plasmid); the LV-MALAT1-vector group (infected with lentivirus over-expressing MALAT1); the LV-CON-shRNA group (infected with lentivirus containing control shRNA); the LV-MALAT1-shRNA group (infected with lentivirus containing MALAT1-shRNA); the LV-MyoD-vector group (infected with lentivirus over-expressing MyoD); the LV-MALAT1 + LV-CON-vector group (infected with lentivirus over-expressing MALAT1 and empty plasmid); the LV-MALAT1-vector + LV-MyoD-vector group (infected with lentivirus over-expressing MALAT1 and MyoD).

All rats from each group were injected with 8 μL lentivirus with a titer of 10^7^ TU/mL via the tail vein [42]. All rats were provided with common feed with were allowed free access to water and maintained on a 12/12 h light/dark cycle.

The AMSCs from the aforementioned SHRs were prepared into a 5.0 × 10^7^ cells/mL cell suspension, which was then seeded in a 6-well plate with 2 mL in each well. When cell confluence reached 40%, the cells were infected with lentivirus and after 24 h, the cells were collected for subsequent experimentation.

### Blood pressure determination

Firstly, the rats were anaesthetized by intraperitoneal injection with pentobarbital (1 mg/kg) [43]. Then, the left common carotid artery was isolated, clipped, and a small hole was made using ophthalmic scissors. Next, a polyethylene tube filled with 1% heparin sodium was inserted into the left common carotid artery, which was then sutured with operative lines, and the bulldog clamp was released. Finally, the MAP and HR values of SD rats and SHRs were recorded using a MP150 multichannel physiologic recorder (BIOPAC systems, Inc., Goleta, CA, US).

### HE staining

The thoracic aorta was quickly isolated from 10 SHRs and 30 SD rats, fixed with 4% paraformaldehyde, paraffin-embedded, and sliced into 4 μm serial sections. After being baked at 60°C for 1 h, the sections were deparaffinized in xylene, then washed with gradient ethanol, and rinsed in water. Then, the sections were stained with hematoxylin for 10 min, and washed with distilled water for 1 min. Next, the sections were differentiated with 1% hydrochloric alcohol for 20 s, and washed with distilled water for 1 min. After being soaked with 1% ammonia for 30 s and washed with water for 1 min, the sections underwent eosin staining for 3 min, and then washed with distilled water for 1 min. Afterward, the sections were dehydrated in gradient ethanol (2 min each time), cleared with xylene two times (5 min each time), and sealed with neutral gum. Finally, the sections were observed under an optical microscope (Olympus, Tokyo, Japan). The experiment was repeated three times.

### Masson staining

The tissue specimens were deparaffinized, hydrated, and sliced into 5 sections. The sections were stained with Ponceau S staining solution for 2 min, soaked with 0.2% glacial acetic acid aqueous solution, 5% phosphoric acid aqueous solution and 0.2% glacial acetic acid aqueous solution for 2 min respectively, and then underwent methyl green staining for 3 min. After being rinsed with water, the sections were color separated using 95% ethanol, dehydrated with ethanol, cleared in xylene, and sealed with neutral balsam. Next, the sections were observed under a 400-fold optical microscope and the deformed area rich in collagen fibers was stained with blue coloration, and the cell matrix was stained with red coloration. At last, 10 visual fields of sections in each rat were randomly selected for photography, and the degree of fibrosis and media thickness was analyzed using the Image J analysis software.

### Evaluation of left ventricular hypertrophy and remodeling

After extraction, the heart, the left ventricle including ventricular septum, and the right ventricle were weighed. Then, the left ventricle tissues were fixed and sliced into 5-μm sections. Subsequently, the sections were stained with hematoxylin and eosin and the cross-section area of the myocardial cells in the free wall of lateral left ventricle (including epicardium and endocardium) was measured and recorded. In addition, the perivascular fibrosis of intramuscular artery and arteriole in Masson-stained sections was also evaluated.

### Western blot analysis

Cells or vascular tissues of the thoracic aorta in rats from each group were collected, and total protein content was extracted with radioimmunoprecipitation assay lysate (Sigma-Aldrich, St Louis, MO, USA). Nuclear protein lysate was prepared using Qproteome Cell Compartment kits (Qiagen, Valencia, CA, USA). The protein concentration was quantified using a bicinchoninic acid protein concentration determination kit according to the instructions. Then, 20 μg of protein sample was separated with 10% sodium dodecyl sulfate polyacrylamide gel electrophoresis, and transferred onto a polyvinylidene fluoride membrane. Next, the membrane was blocked with 5% skimmed milk powder at room temperature for 1 h, rinsed with phosphate buffered saline (PBS) once, and incubated with the following primary antibodies at 4°C overnight: rabbit anti-Fibronectin (ab2413, dilution ratio of 1 : 1000), rabbit anti-collagen I (ab34710, dilution ratio of 1 : 1000), rabbit anti-Bax (ab32503, dilution ratio of 1 : 1000), rabbit anti-Bcl-2 (ab196495, dilution ratio of 1 : 1000), rabbit anti-MyoD (ab203383, dilution ratio of 1 : 1000), and rabbit anti-GAPDH (ab9485, dilution ratio of 1 : 2500). All aforementioned primary antibodies were purchased from Abcam Inc. (Cambridge, UK). After being rinsed with PBS at room temperature for 3 times (5 min each time), the membrane was incubated with the horseradish peroxidase-labeled rabbit anti-immunoglobulin G (IgG) antibody (ab97051, dilution ratio of 1 : 200, Abcam Inc., Cambridge, UK) at 37°C for 1 h. After being rinsed thrice with PBS at room temperature (5 min each time), the membrane was soaked in an enhanced chemiluminescence reaction solution (Pierce, Rockford, IL, USA) at room temperature for 1 min and developed using X-ray. The gray levels of target bands were analyzed using the Image J software. The experiment was repeated 3 times.

### RT-qPCR

Myocardial or thoracic aortic vascular tissues or cells of each group were collected and total RNA content was extracted using Trizol kit (Invitrogen, Carlsbad, CA, USA). In accordance with the instructions of First Strand cDNA Synthesis kit, the first strand of cDNA was synthesized via reverse transcription with the first strand cDNA synthetase (TaKaRa, Tokyo, Japan). The expression of the genes was determined using RT-qPCR with the SYBR Premix Ex Taq kit (TaKaRa, Tokyo, Japan). The instrument employed was the ABI Prism 7500 Fast Real-Time PCR system (Applied Biosystems, Carlsbad, CA, USA). The gene expression was calculated using the 2^-ΔΔCt^ method. GAPDH was regarded as the internal reference. Primer sequences are shown in Table 1.

**Table 1:** Primer sequence for RT-qPCR.

**Gene**	**Primer sequence (5′ - 3′)**
MALAT1	Forward: CCCCTTCATTGACCTCAACT
	Reverse: ATGAGTCCTTCCACGATACC
U1	Forward: TGGGGAGTAGGAGAAGCCAA
	Reverse: TTGCGGGACGTTTTCACAAG
GAPDH	Forward: CTGACATGCCGCCTGGAGA
	Reverse: ATGTAGGCCATGAGGTCCAC

### MTT assay

Cells from different groups were seeded in a 96-well plate at a density of 1 × 10^5^ cells/mL. After 24 h, 150 μL MTT (Invitrogen, Carlsbad, CA, USA) phosphoric acid buffer was added to each well (0.5 mg/mL) after the culture medium was removed, and cultured for another 4 h. Next, the buffer in each well was gently extracted, and 150 μL dimethyl sulphoxide (0.5 mg/mL) was added to each well. The plate was oscillated for 15 min to sufficiently dissolve the crystals in dark conditions. The optical density value of each well was measured using an excitation wavelength of 490 nm was measured.

### BrdU assay

Cells were inoculated in a 96-well plate (density of rate of 1.5 × 10^5^ cells/mL) in a culture dish with a diameter of 35 mL. After 1 day of culture, the cells were then synchronized in culture liquid containing 0.4% fetal calf serum for 3 days to ensure most cells reached the Go phase. Prior to cell culture, the cells were incubated with BrdU (final concentration was 30 μg/L) at 37°C for 40 min. After that, the cells were fixed with methanol/acetic acid for 10 min, incubated with 0.3% H_2_O_2_-methanol for 30 min to inactivate the endogenous oxidase, and blocked with 5% normal rabbit serum, followed by cultivation with formamide and denatured de-natured nucleic acid. Subsequently, the cells were added with primary monoclonal antibody to mouse BrdU (dilution ratio of 1 : 50) and cells added with PBS or serum were regarded as the NC. The total number of cells and the number of BrdU positive cells were counted under 10 high-power visual fields using a microscope (Zeiss, Jena, Germany) and the labeling index was calculated.

### Flow cytometry

After 24 h of infection, the cells were collected and rinsed with precooled PBS thrice. After being re-suspended with 0.3 mL PBS and fixed with 0.7 mL absolute ethyl alcohol, the cells were preserved at -20°C for 24 h and centrifuged at 716 × g for 15 min. After the fixation solution was discarded, the cells were rinsed with PBS twice. Next, cells were added with 120 μL RnaseA (200 μg/mL), and incubated at 37°C for 30 min. Subsequently, the cells were stained with 300 μL of 50 μg/mL Propidium Iodide (PI) staining solution at 4°C for 30 min in dark conditions.

### Fractionation of nuclear/cytoplasmic RNA

Cell particles were lysed using 175 μL/10^6^ cold RLN1 solution (50 Mm Tris-HCl, pH = 8.0; 140 mM NaCl; 1.5 mM MgCl_2_; 0.5% NP-40; 2 mM Vanadyl Ribonucleoside Complex), incubated on ice for 5 min, and centrifuged at 300 g at 4°C for 2 min. The supernatant was cytoplasmic RNA and the remaining particles were nuclear RNA. The fractionation of nuclear and cytoplasmic RNA was conducted using Trizol (15596018, Invitrogen, Carlsbad, CA, USA).

### FISH

Sublocation of lncRNA MALAT1 in VSMCs was analyzed using FISH kits (Roche Applied Science, Mannheim, Germany). Ribo^TM^ LncRNA FISH Probe Mix (Red) was synthesized by RiboBio (Guangzhou, Guangdong, China). The antagonistic LncMALAT1 probe was regarded as the NC. A coverslip was placed on a 6-well plate, and the VSMCs were seeded into the plate and cultured for 24 h. When cell confluence reached 80%, the coverslip was removed. After being rinsed with 1 × PBS, the cells were incubated in 1 mL 4% paraformaldehyde, treated with protease K and glycine, and incubated in 250 μL of prehybridization solution at 42°C for 1 h. After the prehybridization solution was removed, the cells were hybridized with 250 μL hybridization solution containing probes at 42°C overnight, and then rinsed thrice with Phosphate-Buffered Saline/Tween. The nucleus was stained with 6-diamidino-2-phenylindole, and the cells were observed under a 200-fold fluorescence microscope (Olympus, Tokyo, Japan).

### Dual-luciferase reporter assay

The artificially synthesized gene fragments Myogenin, MCK, and SV40 were introduced into the Myogenin-Luc, MCK-Luc, and SV40-Luc report plasmids (Promega Corporation, Madison, WI, USA). The luciferase reporter plasmids were co-transfected with LV-MALAT1-shRNA, LV-MyoD-vector, LV-MALAT1-vector, and LV-MyoD-vector into cultured VSMCs. After 48 h of transfection, the VSMCs were collected and lysed, and the luciferase activity was detected with a Luminometer TD-20/20 detector (E5311, Promega Corporation, Madison, WI, USA) using a dual-luciferase reporter assay system kit (Promega Corporation, Madison, WI, USA). The experiment was repeated three times in each group to obtain the mean value.

### RIP assay

VSMCs were lysed using lysis buffer (25 mM Tris-HCl, pH = 7.4; 150 mM NaCl; 0.5% NP-40; 2 mM ethylenediaminetetraacetic acid;1 mM NaF; and 0.5 mM dithiothreotol) supplemented with RNasin (Takara, Tokyo, Japan) and protease inhibitor. Next, the lysis buffer was centrifuged at 12000 g for 30 min to collect the supernatant. Then, the cells were added with 2 μg antibody Suv39h1 (Millipore, Billerica, MA, USA), and the cells added with isoform IgG (Santa Cruz Biotechnology, CA, USA) magnetic beads served as the controls. After 4-h of incubation at 4°C, the beads were rinsed with washing buffer (50 mM Tris-HCl; 300 mM NaCl, pH = 7.4; 1 mM MgCl 2; and 0.1% NP-40). RNA content was extracted from the magnetic beads using Trizol and the expression of MALAT1 was measured using RT-qPCR.

### RNA pull-down

VSMCs were transfected with 50 nM biotinylated full-length MALAT1 and the corresponding antisense-MALAT1, 48 h after which the cells were collected and rinsed with PBS. Subsequently, the cells were incubated in specific lysis buffer (Ambion, Austin, Texas, USA) for 10 min and centrifuged at 14000 g to collect the supernatant. Protein lysis buffer was then incubated with M-280 streptavidin-biotin and magnetic beads (S3762; Sigma-Aldrich, St Louis, MO, USA) pre-coated with RNase-free bovine serum albumin and yeast tRNA. The beads were incubated at 4°C for 3 h, rinsed with pre-cooled lysis buffer, low-salt buffer, and high-salt buffer. The bound proteins were purified for Western blot analysis.

### ChIP assay

The antibodies employed in this assay included 5 μg antibody to Suv39h1 (Millipore, Billerica, MA, USA), H3K9me3 (Abcam Inc., Cambridge, UK), MyoD (Santa Cruz Biotechnology, CA, USA) or isoform IgG (Santa Cruz Biotechnology, CA, USA). IgG antibody was regarded as the NC. The pulled-down DNA was resuspended in 20 μL water and 1 μL immunoprecipitation samples were subjected to RT-qPCR. The content of amplified DNA was used to present the relative enrichment with input and values captured after normal IgG immunoprecipitation as the controls. The primer sequences of ChIP-PCR were shown in Table 2.

**Table 2:** Primer sequence of ChIP-PCR.

**Gene**	**Sequence (5′ - 3′)**
Myogenin	Forward: GAATCACATGTAATCCACTGGA
	Reverse: TCACACCAACTGCTGGGTG
MCK	Forward: ACCTAGCCCACCTCTCCCTA
	Reverse: AGAGCGAGCTTCTCCTCCAT

### Statistical analysis

Statistical analyses were conducted using the SPSS 21.0 software (IBM Corp. Armonk, N.Y., USA). Measurement data were expressed as mean ± standard deviation. First, the normal distribution and variance homogeneity were measured. If data conformed to normal distribution and homogeneity of variance, comparisons between two groups were analyzed using the unpaired *t-*test, while comparisons among multiple groups were performed with one-way analysis of variance or repeated-measures analysis of variance, with the Tukey’s post hoc tests for multiple pairwise comparisons. If data did not conform to normal distribution or homogeneity of variance, data were analyzed using the rank sum test. A value of *p* < 0.05 indicated statistical significance.
